# The Distribution, Diversity, and Indicator Species of Coral Communities Under the Influence of Environmental Changes in the Subtropical Peninsula of Southern China

**DOI:** 10.1002/ece3.72212

**Published:** 2025-09-30

**Authors:** Dong‐Hai Wu, Li‐Yong Miao, Yong‐Duo Song, Sai Wang, Tuan‐Tuan Wang, Hui‐Long Ou, Jia Xie, Yang Zhang, Cong‐Cong Jin, Wen‐Tong Xia, Naimat Ullah, Kai‐Dian Zhang, Shi‐Quan Chen, Hai‐Long Zhou, Kuan‐Song Wang

**Affiliations:** ^1^ State Key Laboratory of Marine Resource Utilization in South China Sea Hainan University Haikou China; ^2^ Center for Eco‐Environment Restoration Engineering of Hainan Province, School of Ecology Hainan University Haikou China; ^3^ School of Marine Biology and Fisheries Hainan University Haikou China; ^4^ Shenzhen Guanghuiyuan Environment Water Co., Ltd. Shenzhen China; ^5^ Hainan Academy of Ocean and Fisheries Sciences Haikou China; ^6^ School of Life Sciences Hainan University Haikou China

**Keywords:** anthropogenic disturbance, community structure, coral diversity, environmental factors, indicator species, subtropical zone

## Abstract

Coral reefs are among the most biodiverse and economically important marine ecosystems worldwide. Recently, these ecosystems have faced threats and degradation from multiple factors. In particular, coral species living at the edges of subtropical zones are important “barometers” for indicating the global boundaries of coral communities. From 2023 to 2024, we investigated the coral species composition and coverage surrounding the Dapeng Peninsula, which is located at the northern edge of the subtropical zone, and explored the influence of environmental factors on the coral community structure. Fifty‐one species belonging to 20 genera and 13 families were identified in five habitats (conservation, wind‐wave, tourist, fishery, and pristine areas) across three seasons. Owing to rapid urbanization and global warming, the dominant coral species shifted from branching types, which are essential reef‐builders for creating structural substrates, to clumping types, which are more adaptable to environmental pressures. Notably, compared with the first report four decades ago, our current results demonstrated that coral coverage around the Dapeng Peninsula declined at an approximate rate of 0.54% per year from 1983 to 2023. Redundancy correspondence analysis revealed that substrate type, especially rock and dead coral skeletons, nitrogen and phosphorus levels, suspended particulate matter, chemical oxygen demand, and water transparency were key environmental factors that significantly (*p* < 0.05) influenced the distribution of coral species. The lowest diversity of coral communities was recorded in fishery and wind‐wave areas, where intense human disturbance and harsh natural environments limit the survival of sensitive coral species. In contrast, owing to stable hydrological conditions, shallow sunlit water, and diverse substrate types, coral biodiversity was the highest in the pristine habitat. Accordingly, the coral indicator species varied from *Pavona decussata* in the conservation area to *Platygyra carnosus* in the wind‐wave area, to *Montipora peltiformis* and *Leptastrea purpurea* in the tourist area, to *Turbinaria peltata, Porites deformi*, *Goniopora columna* , and *Goniopora lobata* in the pristine area, while no indicator species were found in the fishery area. The distribution and coverage of these indicator species can effectively reflect the extent of human disturbance to local habitats. In the future, it will be important to conduct real‐time monitoring of coral community characteristics around the Dapeng Peninsula to determine environmental changes in subtropical marine zones, providing insights into the response of global coral communities to anthropogenic disturbances and climate change.

## Graphical abstract

The dominant coral species in Dapeng Peninsula changed from branching types to clumping types. Fishing and tourism had significant negative impacts on coral reef development. Water quality and substrate types determined the distribution and structure of coral communities. Nine coral species across different habitat types were selected as indicators to reflect region‐specific environmental changes.

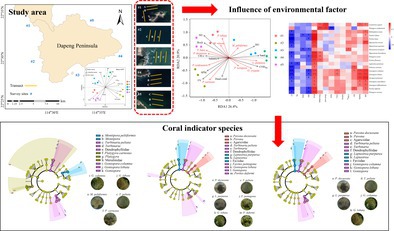

## 1 Introduction

Coral reefs are among the most diverse and valuable ecosystems on Earth. Although coral reefs occupy < 0.1% of the ocean floor, they provide habitats for at least 25% of known marine species, with many reef species yet to be discovered (Fisher et al. 2015). Moreover, coral reefs support a wide range of ecosystem services worldwide, such as fisheries, recreation, tourism, and coastal protection (Moberg and Folke 1999). Coral reefs play crucial roles in the global carbon cycle, encompassing the metabolic processes of organic (e.g., photosynthesis/respiration) and inorganic (e.g., calcification/dissolution) metabolism (Yan et al. 2009). In addition to playing a key role in the carbon cycle (Albright et al. 2015), coral reefs support various economic sectors (e.g., fisheries, tourism, and biotechnology) and provide essential ecosystem services (Fairoz 2022). Furthermore, coral reefs buffer coastal areas from severe weather events, thereby protecting human life, coastal property, and economic activity (Barbier et al. 2011). Given their ecological and economic importance, understanding the factors that influence coral reef health and resilience is critical for their long‐term conservation.

Coral reefs represent distinctive yet vulnerable marine ecosystems and are increasingly threatened by multiple disturbances that are becoming more frequent and severe (Hughes et al. 2013; Pandolfi et al. 2003; Zhao et al. 2010). Owing to human activities (Andrello et al. 2022; Grottoli et al. 2004; Lin et al. 2024) and climate change (Hughes et al. 2018; Klein et al. 2024; Mellin et al. 2022), extensive destruction of coral reefs is occurring worldwide. Since the 1950s, more than half of global coral reefs have disappeared, leading to a decline of over 60% in species diversity (Eddy et al. 2021). Among the multiple factors influencing coral reefs, human modification of coastal land causes numerous environmental problems, including water pollution and habitat degradation through increased sedimentation, nutrient runoff, and physical disturbances (e.g., dredging and shoreline construction), all of which contribute significantly to coral reef decline (Iyiola et al. 2022; Nichols et al. 2018; Wang et al. 2020; Wenger et al. 2015). In addition, global warming has led to increasingly frequent and severe marine heatwaves, which cause thermal stress and widespread coral bleaching (Hughes et al. 2017; Skirving et al. 2019). For example, elevated sea surface temperatures disrupt the symbiotic relationship between corals and zooxanthellae, reducing coral growth, reproduction, and survival (Wooldridge 2013). Therefore, subtropical coral reefs have suffered extensive negative impacts, leading to a pronounced decline in the biodiversity of coral communities (Timmers et al. 2021).

Coral reefs distributed in the northern subtropics, especially near the edge of the Tropic of Cancer, are situated at the poleward limit of coral distribution. As such, they serve as important ‘barometers’ for detecting shifts in the global distribution of coral reefs in response to climate change and ocean warming (Humphreys et al. 2022). Owing to rapid urbanization and coastal development, extensive coral reefs along the coastline of the South China Sea have experienced degradation and are facing unprecedented destruction (Qin et al. 2019; Tkachenko and Soong 2017). In particular, coral reefs in the eastern seas of Shenzhen, an international metropolis in southern China, are near the northernmost limit of the distribution of reef‐building coral in the Indo‐Pacific region. Coral indicator species—defined as organisms whose presence, absence, or abundance reflects specific environmental conditions—can not only reveal the pressures of human activities but also reflect environmental changes (Lindenmayer and Likens 2011; Wang, Gao, et al. 2024, 2021). By monitoring shifts in the distribution and abundance of coral indicator species, researchers can gain insights into the combined impact of environmental factors, helping to formulate conservation efforts and sustainable management practices.

To support the conservation and sustainable management of coral communities in the northernmost tropical regions, this study investigated spatial variations in coral species composition and distribution around the Dapeng Peninsula, Shenzhen. The objectives of this study were to (1) assess coral community composition, diversity, and structural characteristics based on coral cover; (2) investigate the relationships between coral communities and environmental factors; and (3) identify indicator species and evaluate their ecological implications for coral reef conservation. Our findings offer a comprehensive overview of the coral community structure and environmental conditions in the coastal waters of the northern South China Sea. By establishing baseline data for similar subtropical habitats, this research provides a scientific foundation for ecosystem monitoring and contributes to broader efforts in the conservation and management of global coral reef resources.

## 2 Materials and Methods

### 2.1 Study Area

This study was conducted on the Dapeng Peninsula, which is located in Shenzhen City, Guangdong Province, China. It lies in the northern Tropic of Cancer zone, which serves as a transition zone between the northern margin of the Asian tropics and the southern subtropical zone. The area has a southern subtropical marine monsoon climate, with warm temperatures during both spring and winter. The average annual temperature is 22.3°C, with a peak of 36.6°C and a low of 1.4°C, and the annual average wind speed is 2.4 m/s. The climate is characterized by a short dry season and a long wet season influenced by the Pacific Southeast monsoon, with most of the annual rainfall occurring between April and September. The frost‐free period is long, seasonal wind patterns are evident, and climate conditions vary considerably with elevation, especially in terms of temperature gradients. The area is affected by an average of three to four tropical cyclones (e.g., typhoons and rainstorms) from July to September each year. We selected June, October, and February as survey periods to capture representative seasonal climatic features in Shenzhen (e.g., peak rainfall, typhoon activity, and low winter temperatures), and to enable a multidimensional assessment of the adaptive capacity of coral communities under different environmental stresses.

**FIGURE 1:**
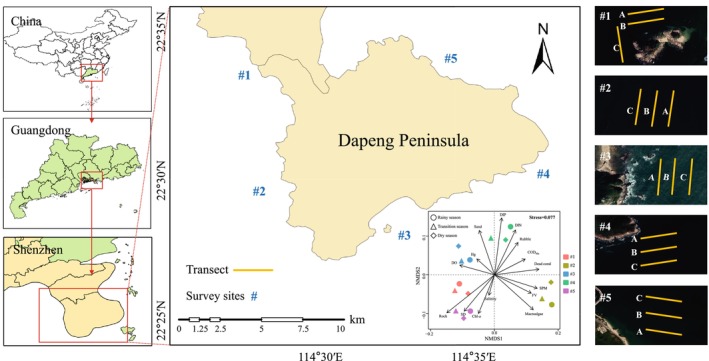
Survey sites and transect lines surrounding the coral communities on the Dapeng Peninsula. Lines A, B, and C at each survey site are three transect lines located at water depths of 3, 6, and 9 m, respectively. In the two‐dimensional ordination space of non‐metric multidimensional scaling (NMDS) plot, the rays represent key environmental factors influencing the site‐specific habitat types. The ray length indicates the strength of environmental factors, and the vertical distance between the sites and the rays reflects the direct influence intensity of environmental factors on the sites: the closer the distance, the greater the influence; the farther the distance, the smaller the influence. FV, flow velocity (m/s); SPM, suspended particulate matter (mg/L); DO, dissolved oxygen (mg/L); COD_Mn_, chemical oxygen demand determined by Mn (mg/L); DIN, dissolved inorganic nitrogen (mg/L); DIP, dissolved inorganic phosphorus (mg/L); Chl‐*a*, chlorophyll‐*a* (μg/L); SD, water transparency (cm); Salinity (‰); and Hg (μg/L). Rock, Rubble, Sand, Macroalgae, and Dead coral are the five substrate types found in coral reefs (expressed as percentages, %).

The study area was selected based on historical records and considerations of the distribution of corals, topography, and water depth. Five typical coral habitat zones surrounding the Dapeng Peninsula were selected. Site #1 was located in a coral conservation area governed by local agencies; site #2 was located in a wind‐wave area; site #3 was located in a tourist area; site #4 was located in a fishery area; and site #5 was located in a pristine area. The non‐metric multidimensional scaling (NMDS) (Figure 1) revealed environmental differences across the five sites, highlighting the variability in habitat conditions (see Tables S1 and S2 for detailed comparisons). Detailed descriptions of the habitat characteristics at each site, including coral cover, environmental conditions, and human activities, are provided in the Supporting Information (Table S3). At each site, three 50‐m‐long transects were established at distinct water depths (3, 6, and 9 m) to capture bathymetric gradients in coral distribution (Figure 1). Surveys were conducted during three distinct hydroclimatic periods: June 2023 (rainy season), October 2023 (transition), and February 2024 (dry season). To ensure diver safety and maintain underwater optical clarity for video transect analysis, field survey was strategically scheduled during rain‐free intervals within each target season. This temporal selection protocol supported the capture of seasonally persistent hydrological stressors (e.g., sustained turbidity from antecedent rainfall) while avoiding acute interference from precipitation during data collection.

### 2.2 Survey Methods

Line intercept transect (Facon et al. 2016; Luthfi and Anugrah 2017): At each survey site, SCUBA‐based underwater observations were conducted along a 50 m transect line laid out on the reef substrate. A handheld GPS device was used to navigate to the predetermined coordinates, where a measuring tape and quadrats were deployed. One diver swam along the transect line at a constant speed of approximately 5 m/min, maintaining a waterproof camera 0.1–0.2 m above the substrate and aligned vertically to the tape. Continuous video footage was recorded to capture the coral community, substrate, and tape reference, with the shooting time of each transect lasting at least 10 min. A second diver followed the videographer to take close‐up photographs of coral species beneath the tape and to collect small samples of rare or difficult‐to‐identify species for taxonomic verification. Additional details of the survey protocol, including equipment setup and daily logistics, are provided in the Supporting Information (Method S1).

### 2.3 Video Transect Interpretation

Video transect analysis was conducted along each 50 m transect using a continuous point‐intercept survey protocol. Data were systematically recorded at 10 cm intervals (*n* = 500 intervals per transect), starting at 0 m and progressing sequentially until the 50 m terminus. Coral assemblages and benthic substrates were quantified through frame‐by‐frame video analysis with the following methodological specifications: (1) Coral species identification—All scleractinian colonies exceeding 2 cm in maximum diameter were taxonomically classified to the species level based on morphological characteristics described in standardized identification guides (Spalding et al. 2001). For specimens exhibiting ambiguous skeletal features (e.g., obscured corallite arrangements or partial tissue necrosis), high‐resolution still images were captured using an Olympus TG‐4 camera (16 MP resolution, 1 cm minimum focus distance) to supplement the video‐based identifications. (2) Live coral coverage calculation—The proportion of live coral cover was determined by dividing the number of intervals containing living coral tissue by the total 500 survey points, expressed as a percentage. Colonies exhibiting > 50% partial mortality or skeletal erosion indicative of mortality events older than 6 months were excluded from the coverage calculations. (3) Substrate characterization—At intervals devoid of epibenthic biota, the underlying substrate was categorized into four classes: consolidated reef rock, unconsolidated rubble (carbonate fragments 2–30 cm in diameter), sand (particles < 2 mm), and dead coral skeleton. Substrate coverage percentages (Table S1) were calculated as the number of intervals containing each substrate type divided by the total number of survey points (*n* = 500). The spatial distribution patterns of both the coral assemblages and the substrate types were subsequently analyzed using geostatistical interpolation techniques to account for small‐scale habitat heterogeneity.

### 2.4 Data Analysis

All the statistical analyses were conducted in R 4.4.0 with the primary packages ‘*picante*’, ‘*vegan*’, ‘*ggplot2*’, and ‘*microeco*’. To quantitatively assess the level of coral community biodiversity in the study area, we used several biodiversity indices commonly applied on the Dapeng Peninsula. These indices include species richness, the Shannon–Wiener diversity index, the Simpson dominance index, and the Pielou evenness index. Richness indicates the number of different species within an area and reflects species diversity. The Shannon–Wiener index accounts for both species richness and the relative coverage of species. The Simpson dominance index highlights the degree to which a few species dominate the community. We also calculated the Pielou evenness index to measure the uniformity of species distributions within coral assemblages across survey sites. These indices were calculated using the following formulae:

(1) $$H^{\prime }=-\sum_{i=1}^{S}\;p_{i}\log_{2}p_{i}$$

(2) $$D=1-\sum_{i=1}^{S}\;{\left(p_{i}\right)}^{2}$$

(3) $$J^{\prime }=H^{\prime }/\log_{2}S$$

where S represents the total number of species; $p_{i}$ represents the proportion of individuals belonging to species i among all individuals (i.e., the ratio of species i coverage to total live coral coverage); $H^{\prime }$ represents the Shannon–Wiener diversity index; D represents the Simpson dominance index; and $J^{\prime }$ represents the Pielou evenness index.

To explore the relative distance and structural differences between coral communities, nonmetric multidimensional scaling (NMDS) analysis was used. NMDS is a multidimensional spatial analysis technique that can simplify complex multidimensional data sets into low‐dimensional space (usually two‐dimensional or three‐dimensional) while trying to maintain the relative distance relationship between samples in the original data. NMDS relies on the rank order of pairwise variable dissimilarities (Bray–Curtis distance in this study) and does not make any underlying distributional assumptions about the data (Wang, Wang, et al. 2021). The analysis was performed using the vegan package in the R language, with the Bray–Curtis distance used as the measure of similarity between samples. The best dimension was determined via cross‐validation. To ensure a robust NMDS solution, we performed 100 random restarts to identify the optimal configuration and assessed the model fit using stress values, with low stress indicating good correspondence to the original distance matrix.

Linear discriminant analysis effect size (LEfSe) is an algorit hm used to discover high‐dimensional indicators that identify taxa by characterizing the differences between two or more biological conditions (Segata et al. 2011). LEfSe emphasizes both statistical significance and biological relevance, enabling researchers to identify discriminative features that are significantly different between biological classes (Wang, Wu, et al. 2024). LEfSe first provides a list of features that are differential among groups with statistical and biological significance, ranks them according to effect size, and then maps the differences to taxonomic trees (Wang et al. 2023). Specifically, the nonparametric Kruskal–Wallis test was used to detect features with significant differences in abundance between categories, and the paired Wilcoxon rank‐sum test was used to assess whether these features also differed consistently across subclasses. Finally, linear discriminant analysis (LDA) was applied to estimate the effect size of each differentially abundant feature, providing an interpretable ranking of the taxa that most contributed to the observed differences.

During transect deployment, key environmental variables (Table S2) (e.g., flow velocity, suspended particulate matter, dissolved oxygen, nutrient concentrations, and heavy metals) were measured using standard protocols. Details of all field and laboratory procedures are provided in the Supporting Information (Method S2). To explore the relationships between environmental variables and coral community structure, we employed redundancy analysis (RDA), a constrained ordination technique suitable for modeling linear species–environment relationships . conducted a detrended correspondence analysis (DCA), which indicated gradient lengths of < 3 for all axes (Figure S1), thereby justifying the use of RDA over a unimodal method such as canonical correspondence analysis (CCA). For efficiency, stepwise forwards selection was used to reduce the number of explanatory variables linearly correlated with the axes in the RDA (Wang et al. 2025, 2021). The non‐collinear variables included the relative coverage of four substrate types (rock, rubble, sand, and dead coral skeletons), suspended particulate matter (SPM, mg/L), water transparency (Secchi depth, cm), macroalgae coverage (%), flow velocity (FV, m/s), dissolved oxygen (DO, mg/L), chemical oxygen demand (COD_Mn_, mg/L), dissolved inorganic nitrogen (DIN, mg/L), dissolved inorganic phosphorus (DIP, mg/L), chlorophyll‐*a* (Chl‐*a*, μg/L), mercury (Hg, μg/L), and salinity (‰). The significance of the RDA axes and individual environmental predictors was assessed via Monte Carlo permutation tests (999 permutations) (Legendre and Legendre 2012). Only environmental variables with statistically significant contributions (*p* < 0.05) were retained in the final model to explain the spatial variation in coral community composition. To further assess the strength of the associations between individual coral taxa and environmental variables, we conducted a Pearson correlation analysis to provide a more in‐depth interpretation of the key environmental factors.

## 3 Results

### 3.1 Coral Species Composition and Relative Coverage

**FIGURE 2:**
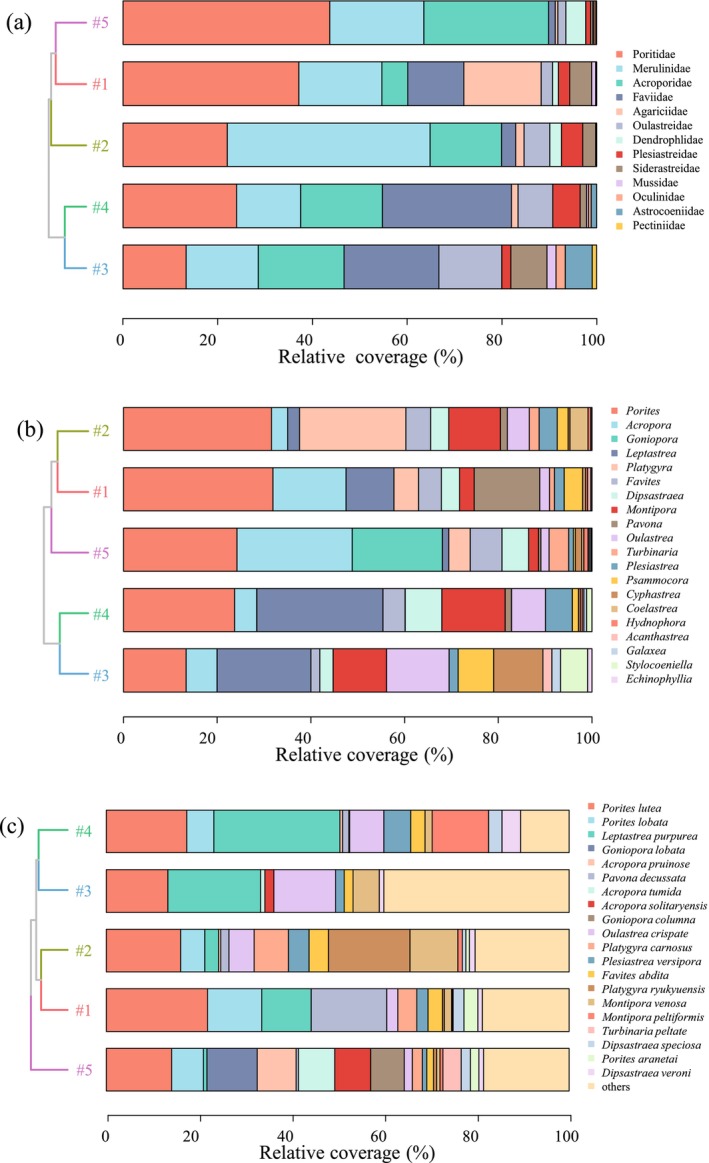
The family, genus, and species compositions of coral communities as reflected by their relative coverage (%) at the five survey sites. The top 20 dominant families, genera, and species of coral communities are shown in (a), (b), and (c), respectively. Site #1: Conservation area; Site #2: Wind wave area; Site #3: Tourist area; Site #4: Fishery area; and Site #5: Pristine area.

A total of 51 coral species from 20 genera and 13 families were identified across the five sites over three seasons (Table S4). Six genera and 18 species of Merulinidae were the most commonly observed corals, accounting for 30.0% and 35.3% of the total genera and species, respectively, followed by Poritidae (2 genera and 8 species) and Acroporidae (2 genera and 7 species). Seasonally, 41, 43, and 45 species were recorded during the rainy, transition, and dry seasons, respectively. Spatially, the number of species ranged from 19 at site #4 to 49 at site #5. The coral coverage (%) at each survey site (Table S5) was as follows: #5 (54.9% ± 5.7%) > #1 (23.9% ± 6.6%) > #3 (14.9% ± 4.7%) > #2 (11.6% ± 5.6%) > #4 (3.0% ± 0.9%). At the family level (Figure 2a), the corals with annual coverages > 5% (averaged across five survey sites and three seasons) included Poritidae (36.4% ± 35.7%), Merulinidae (20.8% ± 15.5%), Acroporidae (19.1% ± 21.8%), and Faviidae (7.9% ± 8.3%), the sum of which accounted for 84.0% of the total coverage. At the genus level (Figure 2b), the corals with annual coverages > 5% included *Porites* (26.6% ± 17.6%), *Acropora* (16.9% ± 19.5%), *Goniopora* (9.3% ± 16.4%), *Leptastrea* (7.5% ± 7.6%), *Platygyra* (6.2% ± 5.2%), *Favites* (5.8% ± 6.3%), *Dipsastraea* (5.2% ± 4.1%), and *Montipora* (5.2% ± 3.2%), the sum of which accounted for 82.5% of the total coverage. The large deviation in *Goniopora* (9.3$ ± 16.4%) was caused by its absence or scarcity at sites #1–#4 but extremely high coverage at site #5. At the species level (Figure 2c), the corals with annual coverages > 3% included *Porites lute* (16.5% ± 7.9%), *Porites lobata* (7.4% ± 4.9%), *Leptastrea purpurea* (7.3% ± 5.7%), *Goniopora columna* (5.5% ± 7.5%), *Acropora pruinosa* (4.4% ± 8.2%), *Pavona decussata* (4.2% ± 4.4%), *Acropora tumida* (4.0% ± 8.9%), and *Acropora solitaryensis* (4.0% ± 8.6%), the sum of which accounted for 60.5% of the total coverage.

### 3.2 Alpha Diversity Indices of Coral Communities

**FIGURE 3:**
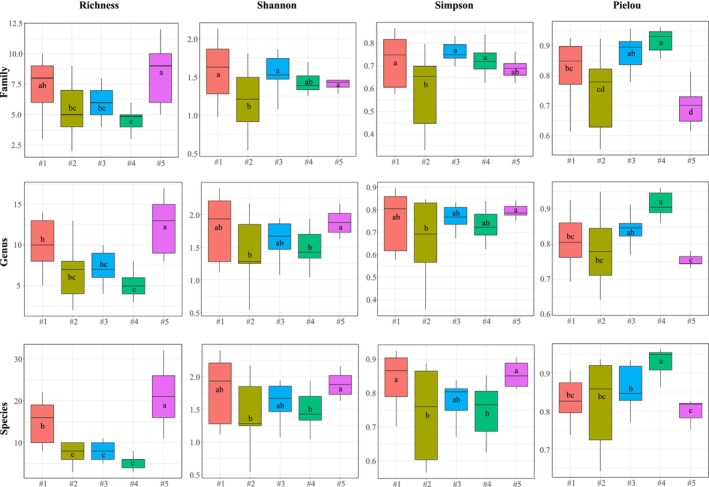
Richness, Shannon–Wiener diversity, Simpson dominance, and Pielou evenness indices of the coral communities at the five survey sites. Different lowercase letters (a–d) above the boxes indicate statistically significant differences among groups (*p* < 0.05). Groups that do not share a common letter are significantly different from each other.

Four indices, including richness, Shannon, Simpson, and Pielou, were used to evaluate the alpha diversity of the coral communities (Figure 3). Regardless of classification level, the highest values of the richness, Shannon, and Simpson indices were recorded at pristine site #5, followed by conservation site #1, both of which had index values significantly (*p* < 0.05) greater than those at wind–wave site #2, tourist site #3, and fishery site #4. The high species richness at sites #5 and #1 may explain the high diversity of the coral communities at these two sites, which were closely associated with the Shannon and Simpson indices. The lowest values of the richness, Shannon, and Simpson indices were recorded at site #4, where the coral communities were affected by intense human activities, such as sailing, diving, and recreation. The Pielou index showed a trend opposite to that of the richness, Shannon, and Simpson indices: the lowest value of the Pielou index was recorded at site #5, whereas the highest values of the other indices were recorded at site #4. The Pielou index value at site #5 was the lowest because of the absolute dominance of *Porites*, *Acropora*, *Turbinaria*, and *Goniopora*, which led to an imbalance in the proportion of the species in the local coral communities. The value of the Pielou index was highest at site #4 because of the extremely low species richness, which led to the lowest value for the denominator of the Pielou index. Notably, the trends in alpha diversity at the family and genus levels were more closely associated with the degree of local human disturbance than those at the species level.

### 3.3 Spatiotemporal Variation in Coral Community Structure

**FIGURE 4:**
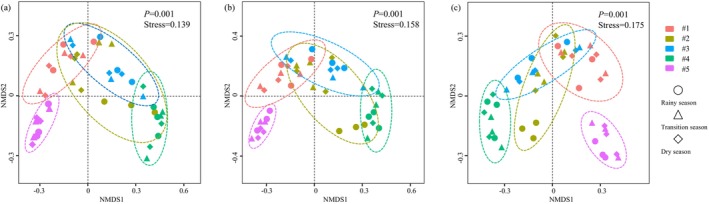
Spatiotemporal variation in the structure of coral communities at the family (a), genus (b), and species (c) levels based on NMDS analysis. The position of each point indicates the similarity between species, while the ellipses around groups represent 95% confidence intervals.

The NMDS results (Figure 4) revealed that the variation in coral community characteristics was more pronounced at the spatial scale than at the temporal scale. Regardless of the classification level, the coral communities at sites #5 and #4 could be clearly distinguished from those at the other sites. The high independence of the coral community structure at site #5 could be attributed to the presence of unique coral species at this site, such as Poritidae*|Goniopora|G. columna*, *G. lobata* , and *G. djiboutiensis* , as well as Dendrophylliidae|*Turbinaria|T* *. peltata* (Table S5). In contrast, given that the coral species that appeared at site #4 were also recorded at the other sites, the high independence of the coral community structure at site #4 could be attributed to the dominance of *Platygyra ryukyuensis* , which had high absolute coverage at site #4. There was some species overlap among sites #1, #2, and #3 because of common coral species with high coverages distributed at these sites, such as Poritidae|*Porites*| *P. lutea* and *P. lobata* , Siderastreidae*|Psammocora|P. superficialis*, and Oulastreidae*|Oulastrea|O. crispate* (Table S5). Notably, compared with the sensitive coral species (e.g., *Goniopora*) distributed only at site #5, the common coral species distributed at sites #1, #2, and #3 were more tolerant of human disturbance. These results indicated that the coral community structure surrounding the Dapeng Peninsula could be classified into 3 levels: sensitive community structure indicating pristine habitats with less disturbance (e.g., site #5), moderately sensitive community structure indicating a certain amount of disturbance (e.g., sites #1–#3), and tolerant community structure indicating strong disturbance (e.g., site #4).

### 3.4 Relationships Between Environmental Factors and Coral Community Structure

**FIGURE 5:**
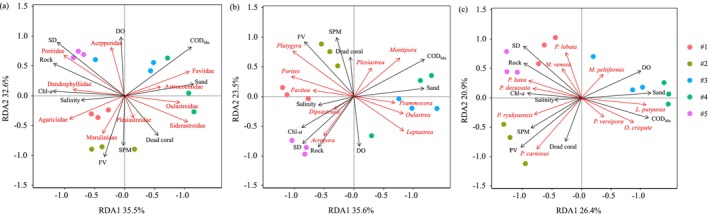
Redundancy analysis (RDA) between the environmental factors and the structure (i.e., relative coverage %) of coral communities, with the relative coverage percentage based on the mean of three transects per survey site. In the two‐dimensional ordination space of RDA plot, the rays represent environmental factors. The ray length indicates the strength of environmental factors, and the vertical distance between the corals and the rays reflects the influence intensity of environmental factors on the corals: the closer the distance, the greater the influence; the farther the distance, the smaller the influence. (a) RDA between environmental factors and family‐level coral coverage, (b) RDA between environmental factors and genus‐level coral coverage, (c) RDA between environmental factors and species‐level coral coverage. DO, dissolved oxygen (mg/L); COD_Mn_, chemical oxygen demand by Mn (mg/L); Chl‐*a*, chlorophyll‐*a* (μg/L); SPM, suspended particulate matter (mg/L); FV, flow velocity (m/s); Sand, sand substrate (%); Rock, rock substrate (%); Dead coral, dead coral substrate (%); Salinity (‰); SD, water transparency (cm).

**FIGURE 6:**
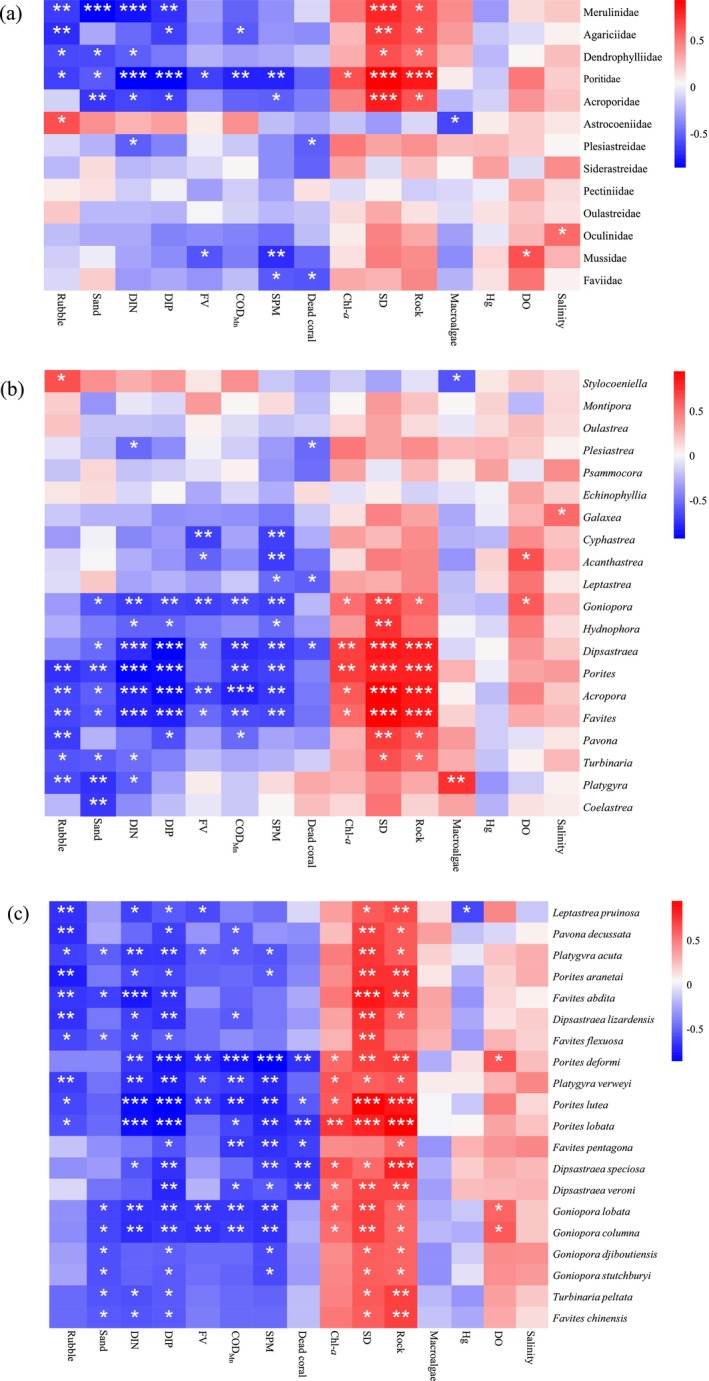
Pearson correlation analysis between the environmental factors and the distributions of coral families (a), genera (b), and species (c) at the five survey sites. FV, flow velocity (m/s); SPM, suspended particulate matter (mg/L); DO, dissolved oxygen (mg/L); COD_Mn_, chemical oxygen demand determined by Mn (mg/L); DIN, dissolved inorganic nitrogen (mg/L); DIP, dissolved inorganic phosphorus (mg/L); Chl‐*a*, chlorophyll‐*a* (μg/L); SD, water transparency (cm); salinity (‰); and Hg (μg/L). Rock, rubble, sand, macroalgae, and dead coral are the five substrate types found in coral reefs (expressed as percentages, %).

The RDA results (Figure 5) revealed that the explanatory power of environmental factors for coral coverage at different taxonomic levels tended to be as follows: family (68.1%) > genus (59.1%) > species (47.3%). Both RDA and Pearson's correlation analysis (Figures 5 and 6) revealed that the environmental factors could generally be divided into three categories: (1) high values of DO, Secchi depth, and dead coral skeleton coverage, which indicated that good water quality and substrate diversity were closely associated with sensitive coral species (e.g., Acroporidae|Montipora| *M. venosa* , Plesiastreidae|Plesiastrea| *P. versipora* , and Astrocoeniidae|Stylocoeniella| *S. guentheri* ); (2) high values of flow velocity, SPM, and rock substrate coverage, which indicated exposed marine conditions and a certain degree of water pollution and were closely associated with moderately sensitive coral species (e.g., Agariciidae|Pavona| *P. decussata* , Oculinidae|Oulastrea|*O*. *crispate*, and Mussidae|Acanthastrea| *A. echinata* ); and (3) high values of COD_Mn_, DIN, DIP, and sand substrate coverage, which indicated severe nearshore human disturbance and were closely associated with tolerant coral species (e.g., Poritidae|Porites| *P. lutea* , Merulinidae|Platygyra| *P. carnosus* , and Siderastreidae|Psammocora| *P. superficialis* ).

### 3.5 Coral Indicator Species Selected by LEfSe

**FIGURE 7:**
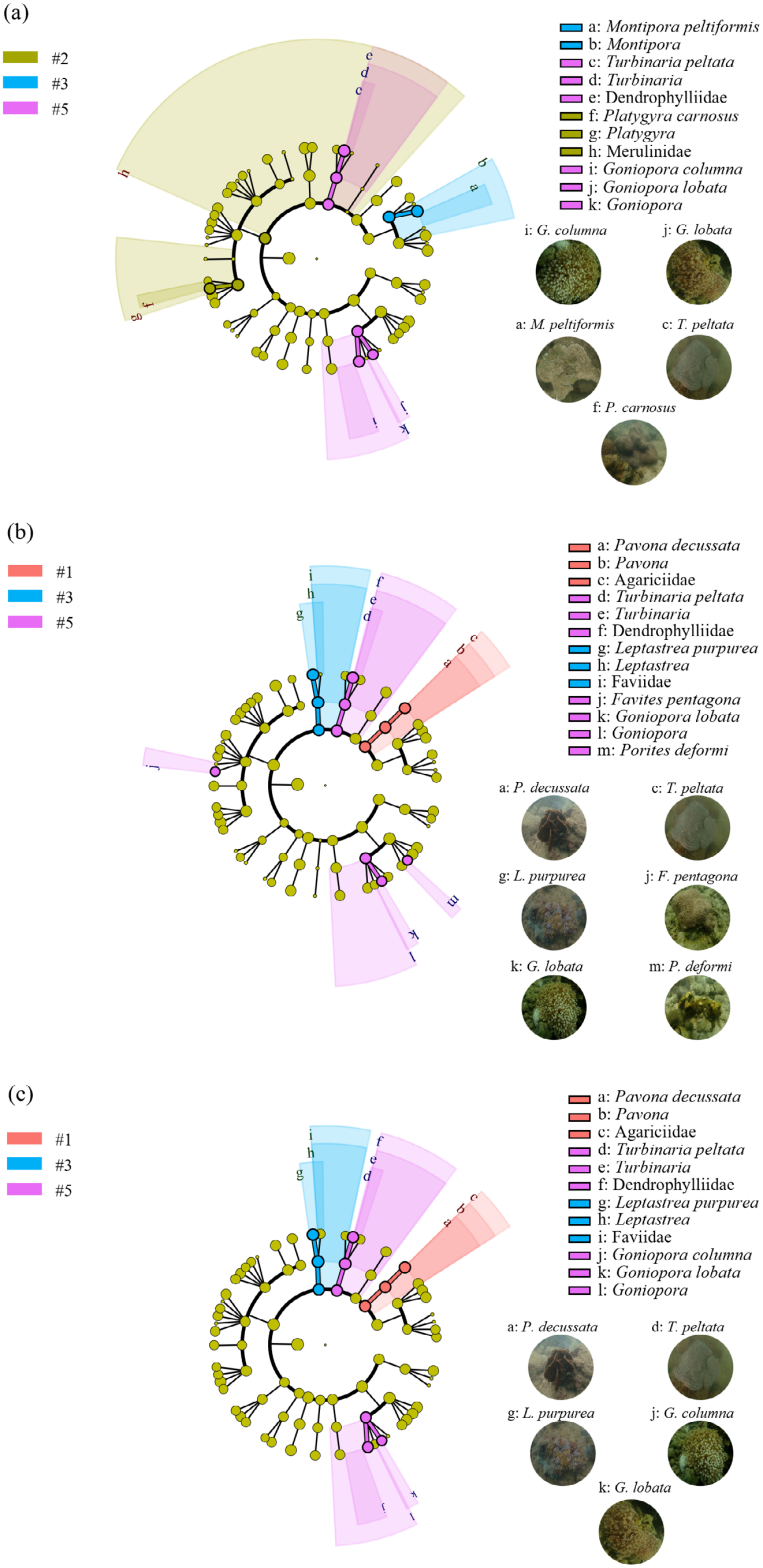
Coral biomarkers selected by LEfSe from the five survey sites around the Dapeng Peninsula. (a) Rainy season; (b) transition season; (c) dry season. The circles represent the classification levels of order, family, genus, and species from the outside to the inside; the size of the fan‐shaped area is proportional to the representativeness of the coral biomarkers. The circular pictures of species represent biomarkers.

Nine coral species belonging to 4 families and 8 genera were selected as indicator species via LEfSe (Figure 7). During the rainy season, 5 indicator species belonging to 2 families and 4 genera were selected (Figure 7a), including *Merulinidae*|*Platygyra*| *P. carnosus* at site #2, *Montipora*| *M. peltiformis* at site #3, and Dendrophylliidae|*Turbinaria*| *T. peltata* , *Goniopora*| *G. columna* , and *Goniopora*| *G. lobata* at site #5. During the transition season, 6 indicator species belonging to 3 families and 4 genera were selected (Figure 7b), including Agariciidae|*Pavona*| *P. decussata* at site #1, Faviidae|*Leptastrea*| *L. purpurea* at site #3, as well as Dendrophylliidae|*Turbinaria*| *T. peltata* , *F. pentagona* , *P. deformis* , and *Goniopora*| *G. lobata* at site #5. During the dry season, 5 species belonging to 3 families and 4 genera were selected (Figure 7c), including Agariciidae|*Pavona*| *P. decussata* at site #1, Faviidae|*Leptastrea*|*L*. *purpurea* at site #3, and Dendrophylliidae|*Turbinaria*| *T. peltata* , *Goniopora*| *G. columna* , and *Goniopora*| *G. lobata* at site #5. Generally, *G. lobata* , *F. pentagona* , *P. deformis*, *G. columna* , and *T. peltata*, which appeared only at site #5, indicated a pristine area with low levels of human disturbance; and *M. peltiformis* and *L. purpurea* , which appeared only at site #3, indicated a tourist area with moderate human disturbance. The presence of *P. decussata* at site #1 indicated a conservation area with human protection. The presence of *P. carnosus* at site #2 indicated the presence of a wind‐wave area with strong climatic effects. Notably, no indicator species were selected by LEfSe at site #4, the fishery area, suggesting that local coral species were not representative enough to indicate the local environment (e.g., water quality or habitat characteristics).

## 4 Discussion

### 4.1 Historical Succession of the Coral Community on the Dapeng Peninsula

In the coastal region of the Dapeng Peninsula, the coral areas are located primarily in Daya Bay (sites #4 and #5) and Dapeng Bay (sites #1, #2, and #3). The first report of coral species and their coverage in Daya Bay dates back to 1983–1984, at which time the coral coverage was approximately 76.6% (Zhang and Zou 1987), and the dominant species belonged to the branching Acroporidae (e.g., *Montipora* and *Acropora*) (Chen et al. 2007). Compared with the survey conducted four decades ago, our current findings demonstrate that coral coverage in Daya Bay has decreased to 55.0%, with the dominant species now belonging to clumping Poritidae (e.g., *Porites*) (Zhao et al. 2022). Accordingly, we can conclude that there has been an approximate rate of decline in coral of 0.54% per year from 1983 to 2023. In addition, the species composition of coral communities has undergone substantial changes—the life history of dominant coral species has shifted from sensitive branching corals, which are significant reef‐building species that create structural substrates, to tolerant clumping corals, which have a strong ability to resist environmental pressures (Darling et al. 2013; Loya et al. 2001).

Owing to rapid urbanization and global warming, coral communities have been subjected to intense anthropogenic disturbance and natural pressures. As a consequence of the high frequency of coral bleaching events, coral communities in the Pacific region underwent strong degradation in 1997–1998, 2010–2011, and 2014–2017 (Lyu et al. 2022). The South China Sea was also affected by global coral bleaching events, especially from 2015 to 2016, when coral communities along the coastline of Guangdong Province experienced strong negative impacts (Qin et al. 2020). For example, large‐scale coral bleaching occurred in the Beibu Gulf (located southwest of the Dapeng Peninsula) because of the high surface temperatures in the northern South China Sea during the summer of 2020 (Chen et al. 2022). The state of the coral communities has oscillated between phases of bleaching and recovery, leading to a gradual adaptation of the coral population structure to the changing environment (Dudgeon et al. 2010). Therefore, the mortality of certain heat‐sensitive corals during bleaching events may account for the degradation of coral communities, driving the transition of dominant corals from sensitive species to tolerant species (Duffy et al. 2016; Eddy et al. 2018).

### 4.2 Effects of Human Activities on Coral Distribution and Diversity

**FIGURE 8:**
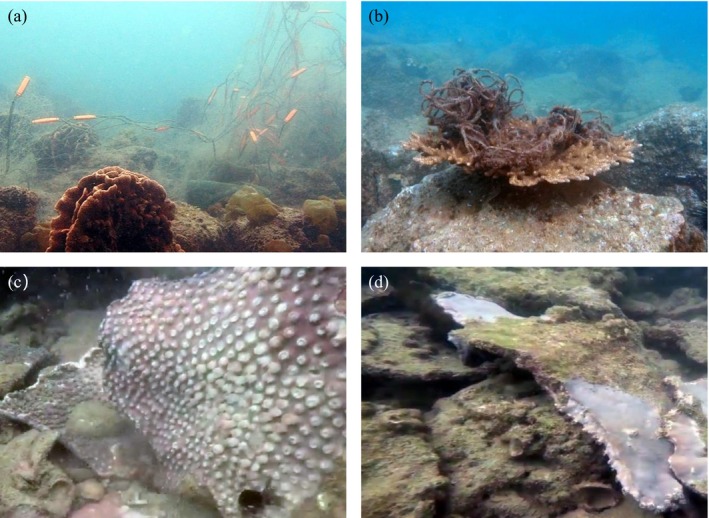
(a, b) Coral entangled in a fishing net, (c) coral before being blown over by high winds, and (d) coral after being blown over by high winds.

Regardless of the morphological classification level, the spatial variation in coral community characteristics could be used to divide the area surrounding the Dapeng Peninsula into three levels of anthropogenic disturbance: sensitive, moderately sensitive, and tolerant coral communities, which indicate low disturbance (e.g., sites #1 and #5), moderate disturbance (e.g., sites #2–#3), and high disturbance (e.g., site #4), respectively. Unlike the western peninsula (e.g., tourism at site #2 and fishing at site #4) with intense human activities and the southern peninsula (e.g., rough waters and strong winds at site #3) with strong hydrological conditions, the pristine habitats at site #5 had good water quality and diverse substrate types; therefore, coral biodiversity was the highest on the eastern side of the peninsula (Castro‐Sanguino et al. 2022). Although the environmental conditions at site #1 were poorer than those at site #5, the coral reserves and nurseries established by conservationists at site #1 effectively protected the coral communities from human disturbance and strong waves (Gardner et al. 2003). In addition, a series of conservation and management measures, such as limiting fishing activities, controlling the number of tourists, delineating diving areas, and prohibiting destructive engineering practices, could help reduce direct damage to coral ecosystems, thereby providing a more stable environment for corals (Halpern et al. 2015). The lowest coral biodiversity was recorded at site #4, which could be attributed to interference from frequent fishing activities. For example, the engines of fishing boats disturb sediments, causing the secondary suspension of particulate matter (e.g., sand or mud) at the water bottom. This disturbance results in increased turbidity of the water and reduced photosynthesis in symbiotic algae. In addition, fishing nets may entangle corals, and when wind and waves occur, the corals entangled with nets are easily pulled over because of strong drag forces from fishing nets with large bearing areas (Figure 8). Therefore, coral communities in disturbed areas should be protected by adopting wind‐wave protection measures and limiting fishing activities.

### 4.3 Monitoring Key Environmental Factors to Protect Coral Community Structure

The spatiotemporal variation in coral community structure under the influence of environmental changes can effectively reflect the health and integrity of marine ecosystems. On the basis of the RDA results, the substrate type (e.g., rock, sand, or dead coral skeleton) and water quality (e.g., DO, COD_Mn_, or SPM) were selected as two sets of environmental variables that significantly influenced the coral community structure. Rock and dead coral skeletons are hard substrates that provide the necessary physical conditions for corals to adhere firmly. In particular, the porous microstructure of a dead coral skeleton supports dense communities of eukaryotic algae, bacteria, and archaea that facilitate organic matter decomposition and nutrient cycling (Sivadas et al. 2020). In contrast, unstable sand substrates and large amounts of SPM may reduce underwater photosynthetic efficiency and even cause coral death due to burial (Bauman et al. 2013). In the wind‐wave area (e.g., site #2), powerful flow fluctuations and wave exposure caused strong mixing of the upper and lower water layers, leading to sharp changes in water temperature, salinity, and SPM concentrations. Such variable environmental conditions may limit coral photosynthesis, pose threats to coral larvae, and even cause coral bleaching (Yu 2019). At tourist site #3, frequent maritime recreational activities (e.g., scuba diving and offshore fishing), inshore sewage discharge, and coastal construction projects caused long‐term and sustained disturbances to coral communities (Zahedi 2008). Nitrogen and phosphorus are key resources for primary producers (e.g., microphytobenthic organisms, macroalgae, and symbiotic algae) in coral ecosystems (D'Croz and O'Dea 2007; Lapointe 1997). The presence of a certain amount of nutrients (e.g., DIN and DIP) could promote the growth of phytoplankton and macroalgae; however, excessive nutrients might cause an outbreak of algae and limit the photosynthetic efficiency of symbiotic algae because of competition for resources (Kuffner et al. 2006).

### 4.4 Ecological Significance of Coral Indicator Species in Marine Protection

The composition of the coral communities around the Dapeng Peninsula is marked by different indicator species, which show more obvious differences at the spatial scale than at the temporal scale. At the spatial scale, the greatest number of coral indicators (5 species) was found at site #5, and the most representative indicator genera were *Turbinaria* and *Goniopora*, which were observed only at this site, significantly distinguishing the local coral community structure from that at the other sites. In the pristine area (e.g., site #5), diverse habitat types, especially extensive dead coral skeletons, high transparency, stable water flow regimes, and suitable nutrient levels facilitated the survival of *Turbinaria* and *Goniopora*, which explained why the greatest number of indicator species was found at this site. The conservation (site #1), tourist (site #3), and wind‐wave (site #2) areas were characterized by low, moderate, and high intensities of disturbance, respectively, corresponding to the indicator species *P. decussata* (lamellar), *M. peltiformis* (branching), *L. purpurea* (submassive), and *P. carnosus* (massed), which presented high, moderate, and low sensitivities, respectively (Table S6) (Khodzori et al. 2024; Montefalcone et al. 2017). The tourist area can be regarded as a transition zone where both tolerant and sensitive coral species exist. In the fishery area (site #4), because intense fishing disturbances, especially trawling and anchoring, lead to direct or indirect (e.g., large amounts of SPM caused by boat engines) coral death (Giglio et al. 2017), no indicator species were found. The spatially heterogeneous distribution of coral indicator species surrounding the Dapeng Peninsula can indicate not only changes in the nearshore marine environment but also anthropogenic disturbance to the structure of coral communities. However, while this method is effective in subtropical coastal areas such as Dapeng Peninsula, local environmental factors, disturbance patterns, and region‐specific coral community composition may limit the general application of indicator species to other regions. In the future, we plan to conduct similar studies in other regions to further demonstrate the comparability of indicator species and their ecological significance.

## 5 Conclusions

Our annual survey of the coral communities surrounding the Dapeng Peninsula revealed that these communities have undergone continuous degradation over the past 40 years, with a shift in dominant species from branching types to clumping types. Harsh physical conditions, such as water depth, low transparency, and strong winds and waves, limit the distribution of coral communities. Fishing and tourism were the primary human activities that strongly negatively affected the corals. In the pristine area on the east peninsula, good water quality and diverse substrate types support the survival of sensitive coral species (e.g., *Goniopora* and *Turbinaria*). From an environmental protection perspective, controlling nutrient concentrations, reducing sewage discharge, and mitigating anthropogenic disturbances are key strategies for coral conservation. Coral community characteristics across different habitat types can be marked by region‐specific indicator species. In the future, long‐term monitoring of these coral indicator species around the Dapeng Peninsula will be crucial for tracking nearshore environmental changes in northern subtropical zones. This monitoring will offer valuable insights into how global coral communities are responding to anthropogenic disturbances and climate change.

## Author Contributions

**Dong‐Hai Wu:** conceptualization (lead), data curation (equal), formal analysis (lead), funding acquisition (equal), investigation (equal), methodology (equal), project administration (equal), writing – original draft (equal). **Li‐Yong Miao:** data curation (equal), formal analysis (equal), investigation (equal), visualization (equal), writing – original draft (equal). **Yong‐Duo Song:** data curation (equal), investigation (equal), software (equal), visualization (equal), writing – original draft (equal). **Sai Wang:** formal analysis (equal), funding acquisition (equal), investigation (equal), project administration (equal), writing – review and editing (equal). **Tuan‐Tuan Wang:** conceptualization (equal), data curation (equal), formal analysis (equal), funding acquisition (equal), methodology (equal), writing – original draft (equal), writing – review and editing (equal). **Hui‐Long Ou:** data curation (equal), investigation (equal), methodology (equal), writing – original draft (equal). **Jia Xie:** methodology (equal), project administration (equal), writing – original draft (equal). **Yang Zhang:** funding acquisition (equal), project administration (equal), writing – original draft (equal). **Cong‐Cong Jin:** conceptualization (equal), data curation (equal), formal analysis (equal). **Wen‐Tong Xia:** investigation (equal), supervision (equal). **Naimat Ullah:** data curation (equal), investigation (equal), methodology (equal). **Kai‐Dian Zhang:** data curation (equal), investigation (equal), methodology (equal). **Shi‐Quan Chen:** data curation (equal), methodology (equal). **Hai‐Long Zhou:** data curation (equal), investigation (equal), methodology (equal). **Kuan‐Song Wang:** data curation (equal), investigation (equal).

## Conflicts of Interest

The authors declare no conflicts of interest.

## Supporting information


**Data S1:** ece372212‐sup‐0001‐supinfo.docx.

## Data Availability

The data that supports the findings of this study is available in the Supporting Information of this article.
