# Supplementary material for: The Distribution, Diversity, and Indicator Species of Coral Communities Under the Influence of Environmental Changes in the Subtropical Peninsula of Southern China
Source: Ecol Evol. 2025 Sep 30;15(10):e72212. doi: 10.1002/ece3.72212 (PMC12483839; doi:10.1002/ece3.72212)
Supplement: Supplementary file 1 — Data S1: ece372212‐sup‐0001‐supinfo.docx. [file ECE3-15-e72212-s001.docx]

**Supporting Information**

**Table S1** Relative coverage (%) of substrate types at the five survey sites around the Dapeng Peninsula

Note: Values are expressed as mean±standard deviation. Different letters (a, b, c, d) next to the values indicate significant differences between groups based on Kruskal-Wallis test. Groups with different letters are significantly different from each other (*P* < 0.05).

| **Site** | **Rock** | **Rubble** | **Sand** | **Macroalgae** | **Dead coral** |  |
| --- | --- | --- | --- | --- | --- | --- |
|  |  |  |  |  |  |  |
| #1 | 57.7±2.5^a^ | 14.1±4.5^d^ | 18.9±2.8^b^ | 1.9±0.4^b^ | 7.5±4.0^c^ |  |
| #2 | 27.5±2.4^c^ | 28.5±0.5^b^ | 10.5±2.4^c^ | 15.5±2.1^a^ | 18.1±2.9^a^ |  |
| #3 | 25.3±4.0^b^ | 32.3±1.7^a^ | 27.7±2.2^b^ | 0^b^ | 14.6±6.4^c^ |  |
| #4 | 38.9±0.7^c^ | 34.7±1.5^ab^ | 18.5±1.0^a^ | 0^b^ | 7.9±2.6^ab^ |  |
| #5 | 59.3±3.6^a^ | 23.4±2.0^c^ | 8.3±1.9^c^ | 0.3±0.6^b^ | 8.7±1.5^bc^ |  |

**Table S2** Physical and chemical parameters of water quality

Note: FV, flow velocity; SPM, suspended particular matter; DO, dissolved oxygen; COD_Mn_, chemical oxygen demand determined by Mn; DIN, dissolved inorganic nitrogen; DIP, dissolved inorganic phosphorus; Chl-*a*, chlorophyll-*a*; SD, water transparency. Values are expressed as mean±standard deviation. Different letters (a, b, c, d) next to the values indicate significant differences between groups based on Kruskal-Wallis test. Groups with different letters are significantly different from each other (*P* < 0.05).

| **Site** | **FV (m/s)** | **SPM (mg/L)** | **DO (mg/L)** | **COD_Mn_ (mg/L)** | **DIN (mg/L)** | **DIP (mg/L)** | **Chl-*a* (μg/L)** | **Hg (μg/L)** | **SD(cm)** | **Salinity(‰)** |  |
| --- | --- | --- | --- | --- | --- | --- | --- | --- | --- | --- | --- |
|  |  |  |  |  |  |  |  |  |  |  |  |
| #1 | 0.24±0.05^cd^ | 7.8±0.4^b^ | 6.2±0.3^ab^ | 1.25±0.06^ab^ | 0.038±0.003^bc^ | 0.029±0.002^cd^ | 4.8±0.6^ab^ | 0.024±0.012^a^ | 380±20^b^ | 30.6±0.5^a^ |  |
| #2 | 0.78±0.03^a^ | 13.3±1.8^a^ | 5.8±0.3^b^ | 1.37±0.06^a^ | 0.043±0.001^b^ | 0.044±0.004^b^ | 3.6±1.2^bc^ | 0.022±0.006^a^ | 344±41^bc^ | 30.9±0.5^a^ |  |
| #3 | 0.45±0.07^b^ | 8.2±0.2^b^ | 6.3±0.4^ab^ | 1.29±0.14^ab^ | 0.044±0.006^b^ | 0.037±0.004^bc^ | 3.9±1.2^abc^ | 0.030±0.012^a^ | 351±13^bc^ | 30.7±0.2^a^ |  |
| #4 | 0.27±0.05^c^ | 8.6±0.6^b^ | 6.3±0.5^ab^ | 1.4±0.09^a^ | 0.070±0.008^a^ | 0.084±0.013^a^ | 2.5±0.5^c^ | 0.020±0.005^a^ | 328±6^c^ | 30.6±0.6^a^ |  |
| #5 | 0.19±0.02^d^ | 7.1±0.1^b^ | 6.7±0.2^a^ | 1.17±0.04^b^ | 0.030±0.005^c^ | 0.025±0.002^d^ | 5.3±0.2^a^ | 0.022±0.01^a^ | 432±24^a^ | 31.1±0.8^a^ |  |

**Table S3** Site-specific coral habitat conditions

| **Site** | **Area type** | **Environmental conditions** | **Coral cover & substrate type** | **Human activities & management** |
| --- | --- | --- | --- | --- |
| #1 | Conservation | Clear, transparent water with low levels of organic pollutants and nutrient salts | High coral cover; substrate predominantly rocky | Coral conservation station operated by volunteers; benefits from active management and continuous protection measures |
| #2 | Wind-wave | Strong wind and wave exposure; elevated concentrations of suspended particulate matter | Low coral cover; substrate mainly composed of gravel, rock, and sand; high coral mortality observed | Limited human activities due to extreme hydrodynamic conditions |
| #3 | Tourist | Moderate wind activity and moderate concentrations of nutrients and organic pollutants | Moderate coral cover; substrate dominated by gravel and sand; high coral mortality observed | Developing tourism; general government-level management with limited coral-specific conservation policies |
| #4 | Fishery | Low water clarity; highest concentrations of nutrients and organic pollutants among all sites | Lowest coral cover; substrate primarily consists of rock and rubble | High intensity of fishing vessel activity; minimal coral management |
| #5 | Pristine | Exceptionally clear water with minimal wind and wave disturbance; lowest levels of nutrients and pollutants | Highest coral cover; substrate predominantly rocky | The area is remote and sparsely populated, with few human activities or targeted coral reef management. |

**Table S4** Coral species recorded at the five survey sites around the Dapeng Peninsula during the rainy, transition, and dry season

Note: “+” indicated that the coral species is recorded during the season

| No. | Family | Genus | Species | Rainy season | Transition season | Dry season |
| --- | --- | --- | --- | --- | --- | --- |
| 1 | Poritidae | *Porites* | *Porites aranetai* | + | + | + |
| 2 |  |  | *Porites lutea* | *+* | *+* | *+* |
| 3 |  |  | *Porites lobata* | *+* | *+* | *+* |
| 4 |  |  | *Porites deformi* | *+* | *+* | *+* |
| 5 |  | *Goniopora* | *Goniopora columna* | *+* | *+* | *+* |
| 6 |  |  | *Goniopora columna* | *+* | *+* | *+* |
| 7 |  |  | *Goniopora stutchburyi* |  | *+* |  |
| 8 |  |  | *Goniopora djiboutiensis* |  | *+* | *+* |
| 9 | Merulinidae | *Platygyra* | *Platygyra ryukyuensis* | *+* | *+* | *+* |
| 10 |  |  | *Platygyra verweyi* | *+* | *+* | *+* |
| 11 |  |  | *Platygyra carnosus* | *+* | *+* | *+* |
| 12 |  |  | *Platygvra acuta* | *+* | *+* | *+* |
| 13 |  | *Cyphastrea* | *Cyphastrea serailia* | *+* | *+* | *+* |
| 14 |  | *Favites* | *Favites abdita* | *+* | *+* | *+* |
| 15 |  |  | *Favites paraflexuosa* | *+* | *+* |  |
| 16 |  |  | *Favites pentagona* | *+* | *+* | *+* |
| 17 |  |  | *Favites complanata* | *+* | *+* | *+* |
| 18 |  |  | *Favites chinensis* | *+* |  |  |
| 19 |  |  | *Favites flexuosa* | *+* | *+* | *+* |
| 20 |  | *Dipsastraea* | *Dipsastraea speciosa* | *+* | *+* | *+* |
| 21 |  |  | *Dipsastraea veroni* | *+* | *+* | *+* |
| 22 |  |  | *Dipsastraea lizardensis* | *+* | *+* | *+* |
| 23 |  |  | *Dipsastraea favus* |  | *+* |  |
| 24 |  |  | *Dipsastraea rotumana* | *+* | *+* | *+* |
| 25 |  | *Hydnophora* | *Hydnophora exesa* |  | *+* | *+* |
| 26 |  | *Coelastrea* | *Coelastrea aspera* | *+* | *+* | *+* |
| 27 | Plesiastreidae | *Plesiastrea* | *Plesiastrea versipora* | *+* | *+* | *+* |
| 28 | Faviidae | *Leptastrea* | *Leptastrea purpurea* | *+* | *+* | *+* |
| 29 |  |  | *Leptastrea pruinosa* | *+* |  | *+* |
| 30 | Agariciidae | *Pavona* | *Pavona decussata* | *+* | *+* | *+* |
| 31 | Acroporidae | *Acropora* | *Acropora digitifera* | *+* | *+* | *+* |
| 32 |  |  | *Acropora solitaryensis* | *+* |  |  |
| 33 |  |  | *Acropora tumida* | *+* |  | *+* |
| 34 |  |  | *Acropora pruinosa* |  |  | *+* |
| 35 |  | *Montipora* | *Montipora peltiformis* | *+* | *+* | *+* |
| 36 |  |  | *Montipora turgescens* | *+* | *+* | *+* |
| 37 |  |  | *Montipora venosa* | *+* | *+* | *+* |
| 38 | Dendrophylliidae | *Turbinaria* | *Turbinaria peltata* | *+* | *+* | *+* |
| 39 |  |  | *Turbinaria reniformis* | *+* | *+* | *+* |
| 40 |  |  | *Turbinaria frondens* | *+* |  | *+* |
| 41 | Siderastreidae | *Psammocora* | *Psammocora superficialis* | *+* | *+* | *+* |
| 42 |  |  | *Psammocora profundacella* |  |  | *+* |
| 43 |  |  | *Psammocora haimeana* | *+* |  |  |
| 44 | Oculinidae | *Galaxea* | *Galaxea fascicularis* | *+* | *+* | *+* |
| 45 |  |  | *Galaxea astreata* | *+* | *+* | *+* |
| 46 | Mussidae | *Acanthastrea* | *Acanthastrea echinata* | *+* |  | *+* |
| 47 |  |  | *Acanthastrea hemprichii* | *+* | *+* | *+* |
| 48 |  |  | *Acanthastrea subechinata* |  |  | *+* |
| 49 | Astrocoeniidae | *Stylocoeniella* | *Stylocoeniella guentheri* | *+* | *+* | *+* |
| 50 | Pectiniidae | *Echinophyllia* | *Echinophyllia aspera* | *+* | *+* | *+* |
| 51 | Oulastreidae | *Oulastrea* | *Oulastrea crispate* |  | *+* | *+* |

**Table S5** Relative coverage (%) of coral species at the five survey sites

**Table S5.1** Relative coverage (%) of coral species at the five survey sites during the rainy season

| Species | #1 | #2 | #3 | #4 | #5 |
| --- | --- | --- | --- | --- | --- |
| *Porites aranetai* | 0.78±0.69 |  |  |  |  |
| *Porites lutea* | 4.16±1.62 |  | 2.43±0.98 | 0.52±0.26 | 10.66±1.82 |
| *Porites lobata* | 1.39±1.97 | 0.26±0.45 | 0.69±1.20 |  | 0.87±0.91 |
| *Porites deformi* |  |  |  |  | 0.52±0.52 |
| *Goniopora columna* |  |  |  |  | 4.51±3.64 |
| *Goniopora columna* |  |  |  |  | 8.84±6.00 |
| *Goniopora stutchburyi* |  |  |  |  |  |
| *Goniopora djiboutiensis* |  |  |  |  |  |
| *Platygyra ryukyuensis* | 0.26±0.26 | 0.26±0.45 |  |  |  |
| *Platygyra verweyi* |  | 0.17±0.30 |  |  | 0.17±0.30 |
| *Platygyra carnosus* | 2.95±3.46 | 2.08±2.93 |  |  | 2.34±0.69 |
| *Platygvra acuta* |  |  |  |  | 0.17±0.30 |
| *Cyphastrea serailia* | 0.17±0.30 | 0.17±0.30 |  | 0.09±0.15 | 0.87±1.28 |
| *Favites abdita* | 0.95±1.05 | 0.09±0.15 | 0.35±0.60 |  | 0.87±1.50 |
| *Favites paraflexuosa* |  |  |  |  |  |
| *Favites pentagona* |  |  |  |  |  |
| *Favites complanata* | 0.09±0.15 |  | 0.43±0.75 |  |  |
| *Favites chinensis* | 0.09±0.15 |  |  |  |  |
| *Favites flexuosa* |  |  |  |  | 0.52±0.90 |
| *Dipsastraea speciosa* | 0.52±0.69 | 0.26±0.26 | 0.26±0.45 |  | 2.86±2.71 |
| *Dipsastraea veroni* | 0.17±0.15 | 0.09±0.15 |  | 0.09±0.15 | 1.04±1.19 |
| *Dipsastraea lizardensis* | 0.09±0.15 |  |  |  | 0.52±0.45 |
| *Dipsastraea favus* |  |  |  |  |  |
| *Dipsastraea rotumana* |  |  |  |  |  |
| *Hydnophora exesa* |  |  |  |  |  |
| *Coelastrea aspera* |  |  |  |  |  |
| *Plesiastrea versipora* | 0.17±0.30 | 0.17±0.30 | 0.78±1.35 |  | 0.17±0.30 |
| *Leptastrea purpurea* | 0.35±0.30 | 0.17±0.15 | 0.35±0.40 | 0.78±0.69 | 0.35±0.40 |
| *Leptastrea pruinosa* | 0.61±0.54 |  | 0.17±0.30 |  | 0.17±0.30 |
| *Pavona decussata* | 2.17±2.21 |  |  |  | 1.21±2.10 |
| *Acropora digitifera* | 0.26±0.45 | 0.26±0.45 |  | 0.35±0.60 | 0.17±0.30 |
| *Acropora solitaryensis* |  |  | 0.09±0.15 | 0.17±0.30 | 0.61±0.54 |
| *Acropora tumida* |  |  |  |  |  |
| *Acropora pruinosa* |  |  |  |  |  |
| *Montipora peltiformis* | 0.09±0.15 |  | 2.60±1.62 |  | 0.35±0.60 |
| *Montipora turgescens* |  |  |  |  |  |
| *Montipora venosa* | 0.61±0.54 |  |  |  |  |
| *Turbinaria peltata* | 0.17±0.30 |  |  |  | 12.57±8.15 |
| *Turbinaria reniformis* |  |  |  |  | 0.17±0.30 |
| *Turbinaria frondens* |  |  |  |  |  |
| *Psammocora superficialis* | 0.17±0.15 | 0.35±0.40 | 0.26±0.45 | 0.17±0.30 | 1.21±0.40 |
| *Psammocora profundacella* |  |  |  |  |  |
| *Psammocora haimeana* |  | 0.26±0.26 |  |  |  |
| *Galaxea fascicularis* |  | 0.09±0.15 |  |  |  |
| *Galaxea astreata* |  |  | 0.26±0.45 |  | 0.09±0.15 |
| *Acanthastrea echinata* |  |  |  |  | 0.09±0.15 |
| *Acanthastrea hemprichii* |  |  |  |  |  |
| *Acanthastrea subechinata* |  |  |  |  |  |
| *Stylocoeniella guentheri* |  |  |  |  |  |
| *Echinophyllia aspera* |  |  |  |  | 0.26±0.45 |
| *Oulastrea crispate* | 0.61±1.05 | 0.78±0.69 | 0.87±0.75 | 0.35±0.40 | 1.56±0.45 |

**Table S5.2** Relative coverage (%) of coral species at the five survey sites during the transition season

| Species | #1 | #2 | #3 | #4 | #5 |
| --- | --- | --- | --- | --- | --- |
| *Porites aranetai* | 0.35±0.60 |  |  |  | 1.39±1.23 |
| *Porites lutea* | 5.55±3.72 | 2.43±1.84 | 2.51±1.33 | 0.17±0.15 | 6.41±3.53 |
| *Porites lobata* | 3.29±4.04 | 0.78±0.94 | 0.95±1.65 |  | 5.29±1.23 |
| *Porites deformi* | 0.09±0.15 |  |  |  | 1.04±0.26 |
| *Goniopora columna* |  |  |  |  | 2.17±0.79 |
| *Goniopora columna* |  |  |  |  | 5.20±4.77 |
| *Goniopora stutchburyi* |  |  |  |  | 0.52±0.90 |
| *Goniopora djiboutiensis* |  |  |  |  | 0.43±0.75 |
| *Platygyra ryukyuensis* |  | 2.95±5.10 |  |  |  |
| *Platygyra verweyi* | 0.35±0.30 |  |  |  | 0.69±0.98 |
| *Platygyra carnosus* |  | 0.26±0.45 |  |  | 1.13±1.73 |
| *Platygvra acuta* | 0.26±0.45 | 0.17±0.30 |  |  | 0.43±0.54 |
| *Cyphastrea serailia* | 0.17±0.30 |  | 0.09±0.15 | 0.43±0.54 | 0.26±0.45 |
| *Favites abdita* | 0.61±0.54 | 0.52±0.69 | 0.52±0.90 | 0.09±0.15 | 0.78±1.35 |
| *Favites paraflexuosa* |  |  |  |  |  |
| *Favites pentagona* | 0.09±0.15 |  |  |  | 0.78±0.26 |
| *Favites complanata* | 0.35±0.40 | 0.09±0.15 |  |  |  |
| *Favites chinensis* |  |  |  |  |  |
| *Favites flexuosa* | 0.17±0.15 | 0.26±0.45 |  |  | 0.52±0.69 |
| *Dipsastraea speciosa* | 0.43±0.40 |  | 0.52±0.45 |  | 0.95±0.40 |
| *Dipsastraea veroni* | 0.26±0.26 | 0.17±0.30 | 0.52±0.52 |  | 0.17±0.30 |
| *Dipsastraea lizardensis* | 0.35±0.40 | 0.26±0.45 |  |  | 0.17±0.30 |
| *Dipsastraea favus* | 0.09±0.15 |  |  | 0.17±0.30 |  |
| *Dipsastraea rotumana* |  | 0.17±0.30 |  |  | 0.26±0.45 |
| *Hydnophora exesa* |  | 0.78±1.35 | 0.09±0.15 |  | 0.17±0.30 |
| *Coelastrea aspera* |  |  |  |  | 0.09±0.15 |
| *Plesiastrea versipora* | 0.78±0.26 | 0.69±0.65 | 1.30±2.03 | 0.09±0.15 | 0.26±0.26 |
| *Leptastrea purpurea* | 3.64±1.35 | 0.26±0.45 | 6.07±6.14 | 0.26±0.00 | 1.47±1.43 |
| *Leptastrea pruinosa* |  |  |  |  |  |
| *Pavona decussata* | 4.68±2.27 | 0.17±0.30 |  |  | 0.26±0.45 |
| *Acropora digitifera* | 0.52±0.45 | 0.17±0.30 | 0.61±1.05 |  |  |
| *Acropora solitaryensis* |  |  |  |  |  |
| *Acropora tumida* |  |  |  | 0.09±0.15 |  |
| *Acropora pruinosa* |  |  |  |  |  |
| *Montipora peltiformis* |  | 0.17±0.30 | 1.65±2.85 |  | 0.87±1.50 |
| *Montipora turgescens* | 0.26±0.26 | 1.82±2.51 | 0.35±0.60 | 0.26±0.26 |  |
| *Montipora venosa* | 0.69±1.20 | 0.26±0.45 |  | 0.26±0.45 | 0.69±1.20 |
| *Turbinaria peltata* |  |  |  |  | 14.91±8.56 |
| *Turbinaria reniformis* |  | 0.17±0.30 |  |  | 0.17±0.30 |
| *Turbinaria frondens* | 0.35±0.60 | 0.26±0.45 |  |  |  |
| *Psammocora superficialis* | 1.56±1.38 | 0.17±0.30 | 0.26±0.45 | 0.26±0.45 | 0.78±0.78 |
| *Psammocora profundacella* |  |  | 0.09±0.15 |  |  |
| *Psammocora haimeana* |  |  |  |  |  |
| *Galaxea fascicularis* |  |  |  |  | 0.17±0.30 |
| *Galaxea astreata* |  |  |  |  |  |
| *Acanthastrea echinata* |  |  |  |  |  |
| *Acanthastrea hemprichii* |  |  |  |  | 0.17±0.30 |
| *Acanthastrea subechinata* |  |  |  |  |  |
| *Stylocoeniella guentheri* |  |  | 0.26±0.45 | 0.26±0.45 |  |
| *Echinophyllia aspera* |  |  |  |  |  |
| *Oulastrea crispate* | 0.09±0.15 | 0.17±0.30 | 1.39±1.23 | 0.17±0.30 | 1.30±1.13 |

**Table S5.3** Relative coverage (%) of coral species at five sampling sites during the dry season

| Species | #1 | #2 | #3 | #4 | #5 |
| --- | --- | --- | --- | --- | --- |
| *Porites aranetai* | 1.04±0.69 | 0.26±0.45 |  |  | 1.65±0.79 |
| *Porites lutea* | 5.98±3.19 | 3.21±1.17 | 2.86±0.78 | 0.52±0.69 | 6.41±3.53 |
| *Porites lobata* | 3.73±3.55 | 0.78±0.94 | 0.95±1.65 |  | 5.29±1.23 |
| *Porites deformi* | 0.26±0.26 |  | 0.35±0.60 |  | 1.04±0.26 |
| *Goniopora columna* |  |  |  |  | 2.43±0.54 |
| *Goniopora columna* |  |  |  |  | 7.02±2.48 |
| *Goniopora stutchburyi* |  |  |  |  |  |
| *Goniopora djiboutiensis* |  |  |  |  | 0.78±0.78 |
| *Platygyra ryukyuensis* |  | 2.95±5.10 |  |  | 0.69±0.65 |
| *Platygyra verweyi* | 0.35±0.30 |  |  |  | 0.69±0.98 |
| *Platygyra carnosus* |  | 0.26±0.45 |  |  | 1.13±1.73 |
| *Platygvra acuta* | 0.26±0.45 | 0.17±0.30 |  |  | 0.43±0.54 |
| *Cyphastrea serailia* | 0.17±0.30 |  | 0.09±0.15 | 0.43±0.54 | 0.26±0.45 |
| *Favites abdita* | 0.69±0.65 | 0.87±0.54 | 0.52±0.90 | 0.09±0.15 | 1.56±0.69 |
| *Favites paraflexuosa* |  |  |  |  |  |
| *Favites pentagona* |  |  | 0.35±0.60 |  | 1.04±0.52 |
| *Favites complanata* | 0.35±0.40 | 0.09±0.15 |  |  |  |
| *Favites chinensis* |  |  |  |  |  |
| *Favites flexuosa* | 0.69±0.98 | 0.26±0.45 |  |  | 0.95±0.60 |
| *Dipsastraea speciosa* | 0.69±0.65 |  | 0.52±0.45 |  | 1.04±0.26 |
| *Dipsastraea veroni* | 0.26±0.26 | 0.17±0.30 | 0.52±0.52 |  | 0.61±0.54 |
| *Dipsastraea lizardensis* | 0.35±0.40 | 0.26±0.45 |  |  | 0.35±0.30 |
| *Dipsastraea favus* |  |  |  |  |  |
| *Dipsastraea rotumana* |  | 0.17±0.30 |  |  | 0.26±0.45 |
| *Hydnophora exesa* | 0.35±0.60 | 0.26±0.45 | 0.09±0.15 |  | 0.17±0.30 |
| *Coelastrea aspera* |  | 0.78±1.35 |  |  | 0.43±0.15 |
| *Plesiastrea versipora* | 0.78±0.26 | 0.69±0.65 | 1.30±2.03 | 0.09±0.15 | 0.26±0.26 |
| *Leptastrea purpurea* | 3.64±1.35 | 0.61±0.30 | 6.07±6.14 | 0.78±0.26 | 3.03±1.17 |
| *Leptastrea pruinosa* | 0.26±0.45 |  |  |  | 0.17±0.30 |
| *Pavona decussata* | 4.85±2.57 | 0.43±0.40 | 0.17±0.30 |  | 0.35±0.40 |
| *Acropora digitifera* | 0.52±0.45 | 0.17±0.30 | 0.61±1.05 |  | 0.35±0.60 |
| *Acropora solitaryensis* |  |  |  |  |  |
| *Acropora tumida* |  |  |  |  |  |
| *Acropora pruinosa* |  | 0.17±0.30 |  |  | 0.26±0.45 |
| *Montipora peltiformis* |  | 0.17±0.30 | 1.65±2.85 |  |  |
| *Montipora turgescens* | 0.69±1.20 | 0.26±0.45 |  | 0.26±0.45 |  |
| *Montipora venosa* | 0.26±0.26 | 1.82±2.51 | 0.35±0.60 | 0.26±0.26 | 0.26±0.45 |
| *Turbinaria peltata* |  |  |  |  | 16.47±7.33 |
| *Turbinaria reniformis* |  | 0.17±0.30 |  |  | 1.04±1.38 |
| *Turbinaria frondens* | 0.35±0.60 | 0.26±0.45 |  |  | 0.26±0.45 |
| *Psammocora superficialis* | 1.56±1.38 | 0.17±0.30 | 0.26±0.45 | 0.26±0.45 | 1.47±0.65 |
| *Psammocora profundacella* |  |  |  |  |  |
| *Psammocora haimeana* |  |  |  |  |  |
| *Galaxea fascicularis* |  |  |  | 0.17±0.30 | 0.17±0.30 |
| *Galaxea astreata* | 0.17±0.30 |  |  |  | 0.17±0.30 |
| *Acanthastrea echinata* |  |  | 0.17±0.30 |  | 0.17±0.30 |
| *Acanthastrea hemprichii* | 0.09±0.15 |  |  | 0.17±0.30 | 0.17±0.30 |
| *Acanthastrea subechinata* | 0.52±0.52 |  |  |  |  |
| *Stylocoeniella guentheri* |  |  | 0.26±0.45 | 0.26±0.45 | 0.09±0.15 |
| *Echinophyllia aspera* |  |  |  | 0.09±0.15 | 0.09±0.15 |
| *Oulastrea crispate* | 1.04±0.26 | 0.95±0.40 | 1.04±0.26 | 0.69±0.15 | 2.25±0.54 |

**Table S6** Sensitivity classification of coral

| **No.** | **Genus** | **Sensitivity level** | **Clarification** |
| --- | --- | --- | --- |
| 1 | *Acropora* | high | Branching morphology; fast-growing but fragile; highly susceptible to bleaching with poor recovery |
| 2 | *Montipora* | high | Short-branched or encrusting; epiphytic growth form; thermally sensitive |
| 3 | *Galaxea* | high | Agglomerated to submassive colonies; highly sensitive to light and thermal stress |
| 4 | *Porites* | low | Massive to submassive forms with smooth surfaces and thick tissue; widely recognized as highly thermally tolerant |
| 5 | *Leptastrea* | low | Encrusting to submassive morphology; generally considered heat-tolerant |
| 6 | *Echinophyllia* | low | Blade-like growth form; typically occurs in deeper waters; low thermal sensitivity |
| 7 | *Cyphastrea* | moderate | Encrusting morphology; moderate thermal tolerance and recovery potential |
| 8 | *Favites* | moderate | Massive to submassive forms with hard skeletons and thick tissue; moderately heat sensitive |
| 9 | *Dipsastraea* | moderate | Massive to submassive growth; moderate sensitivity; capable of good recovery |
| 10 | *Platygyra* | moderate | Brain coral morphology with continuous, meandering skeletal valleys; moderate thermal response |
| 11 | *Pavona* | moderate | Foliose morphology with thin, undulating edges; moderate thermal sensitivity |
| 12 | *Plesiastrea* | moderate | Encrusting to massive forms; small, tightly arranged corallites; moderate thermal tolerance |
| 13 | *Coelastrea* | moderate | Submassive morphology; moderately tolerant to thermal stress |
| 14 | *Hydnophora* | high | Massive to foliose forms; sensitive to elevated temperature due to thin tissue structure |
| 15 | *Goniopora* | high | Massive and columnar forms; long tentacles increase environmental sensitivity |
| 16 | *Psammocora* | moderate | Encrusting to submassive corals; stable under mild heat stress; moderately sensitive |
| 17 | *Acanthastrea* | moderate | Massive growth with large polyps; moderate heat resistance and recovery ability |
| 18 | *Stylocoeniella* | moderate | Encrusting to submassive forms; dense and uniform skeleton; exhibits moderate thermal tolerance |
| 19 | *Turbinaria* | moderate | Massive or foliose morphology; thick tissue and firm skeleton; high thermal tolerance |
| 20 | *Oulastrea* | moderate | Encrusting form; moderate thermal sensitivity; relatively tolerant under heat stress |

**Method S1** Detailed underwater survey protocol

To optimize data quality, all surveys were conducted between 9:00 and 11:00 a.m. daily, when sea conditions were generally calm and underwater visibility was high. Equipment including waterproof cameras, measuring tapes, and quadrats were transported to the survey sites by boat. Upon arrival, divers used handheld GPS units to navigate to specific coordinates. A 50-meter measuring tape was then laid out along the reef substrate to serve as the transect line. One diver conducted continuous video recording along the transect, swimming at a consistent speed (~5 m/min) while holding the camera parallel to the reef at a height of 0.1–0.2 m. Care was taken to avoid camera shaking and maintain vertical alignment with the tape. The video captured the full length of the transect and surrounding benthic organisms, with a minimum duration of 10 minutes per transect. A second diver followed behind, photographing coral species in detail and collecting small samples of rare or ambiguous corals to support species identification in the laboratory. All procedures followed local conservation and ethical guidelines.

**Method S2** Monitoring methods for physicochemical parameters of water quality

During the deployment of the sampling transects, environmental variables were concurrently measured, while water samples that could not be analyzed on-site were collected and subsequently analyzed in the laboratory. All field measurements and laboratory analyses were carried out according to internationally recognised standard protocols, with minor adaptations noted below. Flow velocity (FV): Measured by a 600 kHz ADCP (iFlow RP1200, Hi-Target, China) in bottom-tracking mode (0.5 m bins; 10-min averages). Suspended particulate matter (SPM): Triplicate 1L samples were filtered through pre‑combusted, pre‑weighed GF/F filters (0.7 µm). Filters were rinsed with ammonium‑formate, dried (105℃) and re‑weighed; MDL = 0.2 mg/L. Dissolved oxygen (DO): Submerge the YSI (YSI Incorporated, USA) Water Quality Analyzer's sensor in water, avoid touching the bottom or obstacles, and record the data when the value stabilizes. Permanganate index (COD_Mn_): Acidic KMnO₄ digestion (ISO 8467) with oxalate titration. Dissolved inorganic nitrogen (DIN). Filtered samples (0.45 µm) were analysed on a segmented‑flow auto‑analyser (SEAL AA3 HR). DIN = NO₃⁻ + NO₂⁻ (Cd–Cu reduction) + NH₄⁺ (indophenol); precision ± 2%. Dissolved inorganic phosphorus (DIP): Orthophosphate was measured spectrophotometrically (molybdenum‑blue, ascorbic reduction). Detection limit = 0.03 µmol L⁻¹; duplicates within 5 %. Chlorophyll‑a (Chl‑*a*): Acetone extraction with grinding, fluorometric quantification (ISO 10260). Mercury (Hg): Total dissolved Hg was quantified by cold‑vapour atomic fluorescence (Tekran 2600; US EPA 1631E). Procedural blanks < 0.5 ng/ L; certified‑reference recoveries 97% ± 4%. Water transparency (SD): The Seth's disk is slowly sunk into the water, until the disk surface pattern just disappeared, record the depth at this time (cm) as the transparency value, and the average value was taken by repeating the process three times. Salinity: CTD-derived (SBE 37) PSS-78 values verified against IAPSO standards. All sensors were inter‑calibrated and all analyses quality‑controlled with certified standards to ensure data traceability.

**Figure S1**

**Figure S1** Detrended Correspondence Analysis (DCA) of coral community structure across five survey sites
